# Isolation and characterization of novel mutations in the pSC101 origin that increase copy number

**DOI:** 10.1038/s41598-018-20016-w

**Published:** 2018-01-25

**Authors:** Mitchell G. Thompson, Nima Sedaghatian, Jesus F. Barajas, Maren Wehrs, Constance B. Bailey, Nurgul Kaplan, Nathan J. Hillson, Aindrila Mukhopadhyay, Jay D. Keasling

**Affiliations:** 1 DOE Joint BioEnergy Institute, 5885 Hollis Street, Emeryville, CA 94608 USA; 2 Biological Systems & Engineering Division, Lawrence Berkeley National Laboratory, Berkeley, CA 94720 USA; 3 Department of Plant and Microbial Biology, University of California, Berkeley, CA 94720 USA; 4 DOE Agile BioFoundry, Emeryville, CA 94608 USA; 5 Department of Bioengineering, University of California, Berkeley, CA 94720 USA; 6 Department of Chemical and Biomolecular Engineering, University of California, Berkeley, CA 94720 USA; 7 The Novo Nordisk Foundation Center for Biosustainability, Technical University of Denmark, Denmark, Building 220 Kemitorvet, 2800Kgs Lyngby, Denmark

## Abstract

pSC101 is a narrow host range, low-copy plasmid commonly used for genetically manipulating *Escherichia coli*. As a byproduct of a genetic screen for a more sensitive lactam biosensor, we identified multiple novel mutations that increase the copy number of plasmids with the pSC101 origin. All mutations identified in this study occurred on plasmids which also contained at least one mutation localized to the RepA protein encoded within the origin. Homology modelling predicts that many of these mutations occur within the dimerization interface of RepA. Mutant RepA resulted in plasmid copy numbers between ~31 and ~113 copies/cell, relative to ~5 copies/cell in wild-type pSC101 plasmids. Combining the mutations that were predicted to disrupt multiple contacts on the dimerization interface resulted in copy numbers of ~500 copies/cell, while also attenuating growth in host strains. Fluorescent protein production expressed from an arabinose-inducible promoter on mutant origin derived plasmids did correlate with copy number. Plasmids harboring RepA with one of two mutations, E83K and N99D, resulted in fluorescent protein production similar to that from p15a- (~20 copies/cell) and ColE1- (~31 copies/cell) based plasmids, respectively. The mutant copy number variants retained compatibility with p15a, pBBR, and ColE1 origins of replication. These pSC101 variants may be useful in future metabolic engineering efforts that require medium or high-copy vectors compatible with p15a- and ColE1-based plasmids.

## Introduction

Metabolic engineering of bacterial hosts relies heavily on the heterologous expression of proteins from plasmids that replicate separately from the host chromosome. In order to achieve pathway balance, researchers use a variety of genetic control systems, including variable origins of replication, to control expression level^1^. Changing the origin of replication of a plasmid provides a convenient means of controlling the magnitude of expression of a heterologous pathway, and can dramatically change the performance of the engineered system within a host^2,3^. Plasmids with compatible origins of replication allow metabolic engineers to divide pathways into multiple parts able to be maintained at different copy numbers, thereby allowing for extra flexibility when trying to ensure maximum flux through a pathway^3^. Having a library of well characterized, orthogonal origins with different copy numbers therefore enables engineers to rapidly alter the characteristics of pathways in pursuit of achieving high product titers^1^.

The pSC101 origin is a low-copy, narrow host-range origin that is often used in synthetic biology and metabolic engineering^1^. Low-copy vectors offer many advantages for metabolic engineering, such as low metabolic burden on the host, tight control of gene expression, and segregational stability^4^. The pSC101 origin regulates its copy number in part through a mechanism mediated by the replication initiator protein RepA^5,6^. As a monomer, RepA initiates plasmid replication by binding to one of the many iterons within the origin^7^. This RepA-iteron complex can also associate with other RepA-iteron complexes in nearby plasmids, repressing replication of both vectors^5^. This “handcuffing” process acts as an effective negative control to limit replication of pSC101 to roughly ~5 copies/cell. While multiple groups have isolated RepA or RepA homolog mutants that increase the copy number of their cognate plasmid, only two have been rigorously characterized^8,9^. Substituting E93 of RepA for either lysine or arginine resulted in an increased copy number from ~5 copies/cell to ~30 and ~240 copies/cell, respectively^10^. The authors hypothesized that these mutations disrupted the ability of RepA to dimerize, and hence the ability of the protein to control the copy number. The authors further demonstrated that these mutant origins were stable over many generations, and still compatible with ColE1 plasmids. While the authors created cloning vectors based on the high-copy origin variants, they did not rigorously characterize the effects that these elevated copy numbers had on heterologous gene expression or host growth. Deeper characterization of these altered origins may give greater insight into their usefulness as tools for synthetic biology and metabolic engineering.

In a recent paper, we described the development of a transcription factor based biosensor for industrially important lactams, mediated by the *araC*-family protein ChnR^11^. In an attempt to select for mutations that increased the sensitivity of this system to a series of potential ligands, we isolated multiple mutations in the pSC101 origin of replication localized to the *repA* gene that increased copy number. Many of these mutations were distinct from mutations that have previously been described to increase the copy number of the pSC101 origin or other plasmids replicated via RepA homologs. In this study, we provide insight into the function of some of these mutated loci within the *repA* gene, characterize the effect that the resulting mutated origins have on a protein expressed from cognate plasmids, and determine if multiple mutations can be combined additively to affect copy number.

## Results

### Isolation of pSC101 origin mutants that result in higher copy number

**Figure 1:**
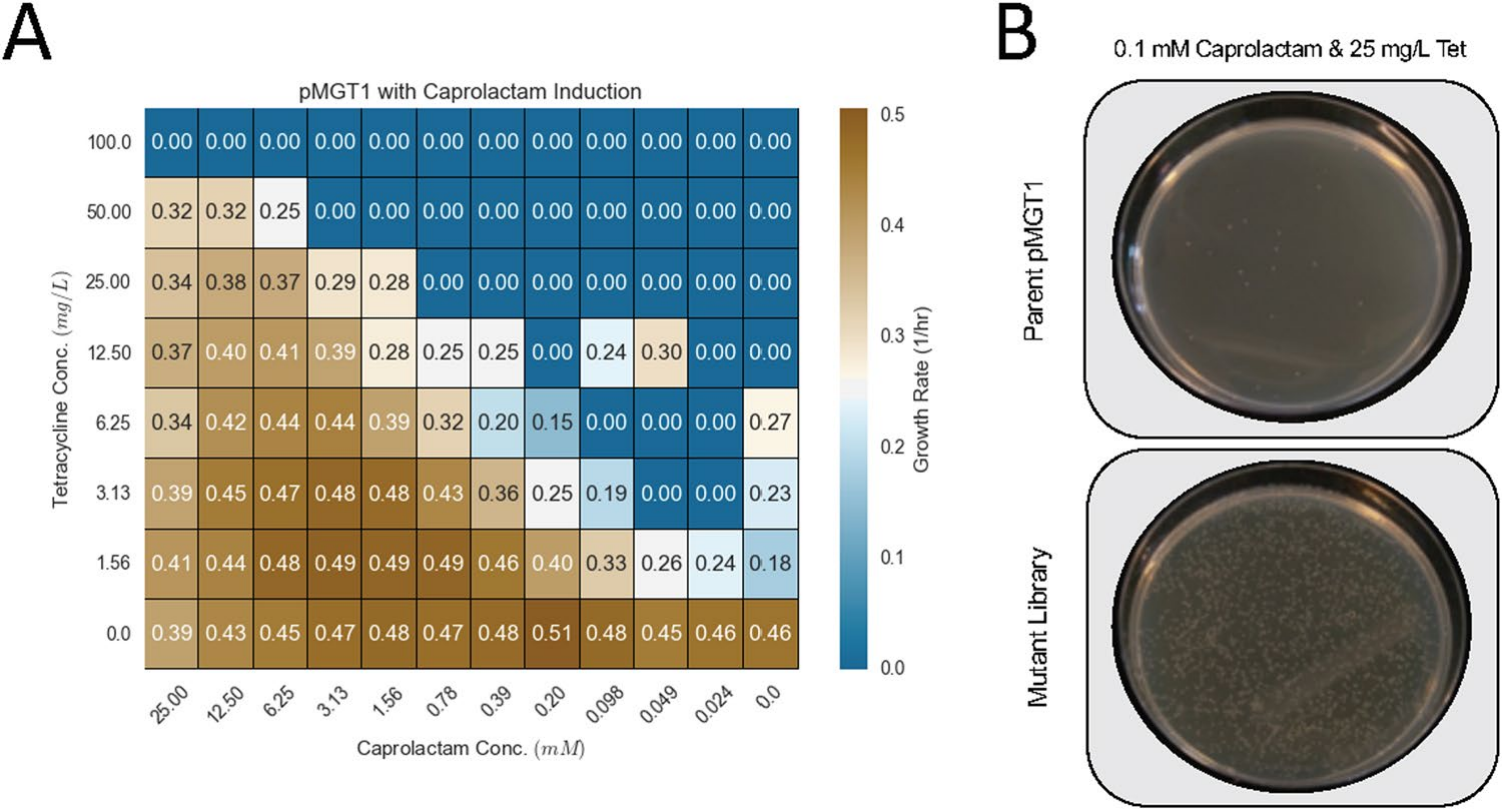
Selection for pMGT1 mutants with greater sensitivity. (**A**) Checkerboard assay showing growth rate of *E*. *coli* harboring pMGT1 challenged with increasing concentrations of tetracycline as a function of increasing concentrations of the inducer caprolactam. (**B**) Results from plate selections of the mutant pMGT1 library plated on LB agar with 0.1 mM caprolactam and 25 mg/L tetracycline compared to *E*. *coli* harboring unmutated parent pMGT1.

Previous work has demonstrated that ChnR may be a useful biosensor for the detection of a myriad of industrially relevant compounds, such as valerolactam and caprolactam^11,12^. While ChnR can detect these molecules, the detection limits are well above what can currently be produced via metabolic engineering of microbial cells^13^. To improve the sensitivity of this transcription factor, we created a system that allowed us to select for ChnR activation through the expression of tetracycline resistance. Previously, tetracycline resistance has been demonstrated to be an excellent reporter to develop selection-based screens for bacterial transcription factors^14^. Plasmid pMGT1 (Supplementary Figure 1) controls the expression of *tetA* via ChnR, which can be activated through induction via caprolactam or other ligands in a dose-dependent manner (Fig. 1A). To generate genetic diversity within this system we created a mutagenized plasmid library by serial passaging of pMGT1 in the commercial *E*. *coli* mutator strain XL1-Red. After the mutated plasmid library was isolated and retransformed into *E*. *coli* DH10B, we used an agar plate-based checkerboard assay to identify mutants that were more sensitive to four potential ChnR ligands: caprolactam, bromocyclohexane, γ-nonalactone, and δ-undecalactone (Supplementary Figure 2). For all putative ligands, the mutagenized library had a higher number of surviving colonies on agar plates supplemented with lower levels of inducers and higher levels of tetracycline than the unmutagenized parent plasmid (Fig. 1B).

**Figure 2:**
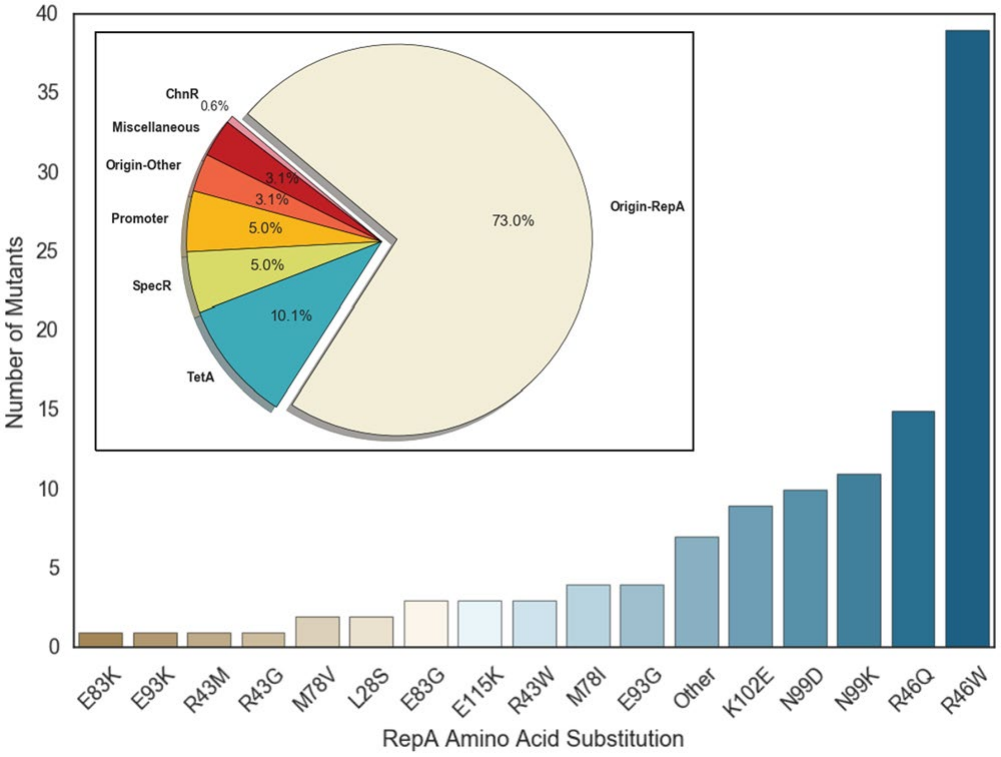
Distribution of mutants obtained from plate-based selections. Inset: Distribution of locations of mutations within the plasmid pMGT1. Histogram showing the frequency of selected amino acid substitutions found within RepA in re-sequenced plasmids.

Thirty plasmids from each ligand were isolated and fully re-sequenced to identify potential mutations. Of the 120 plasmids prepared, 114 were successfully re-sequenced. Within these 114 plasmids, 112 contained mutations relative to wild-type pMGT1 at 59 unique genetic loci. Interestingly, 73% of all mutations were localized within the RepA protein coding sequence of the pSC101 origin, while only 0.6% of the mutations were in *chnR* itself (Fig. 2 inset). Upon further inspection, every plasmid that contained a mutation had at least one mutation within *repA*. Despite the small number of mutations identified within *chnR* itself, the abundance of mutations within *repA* was remarkable. Within our re-sequenced plasmids, we identified 116 mutations in *repA* that corresponded to 22 unique amino acid substitutions at 14 unique amino acid sites. Of the 22 unique amino acid substitutions, 13 occurred more than once (Fig. 2). Forty-six percent of all *repA* mutations occurred at R46, while 18% of all mutations occurred at N99. Overall, 88% of all mutations in *repA* resulted in a substitution of a charged amino acid. Previous reports have shown that mutations shown to increase the copy number of the pSC101 origin are often the result of amino acid substitutions that putatively disrupt dimerization of the RepA protein^9^. A higher copy origin would explain the increased ligand sensitivity found in our plate-based selection, as well as the apparent bias towards charged amino acid mutations, which may participate in protein-protein interactions. Therefore, we hypothesized that many of the amino acid positions that we identified contribute to the dimerization– and corresponding copy number regulation– of the pSC101 origin.

### Isolated mutations in the RepA protein are predicted to lie within the dimerization interface

**Figure 3:**
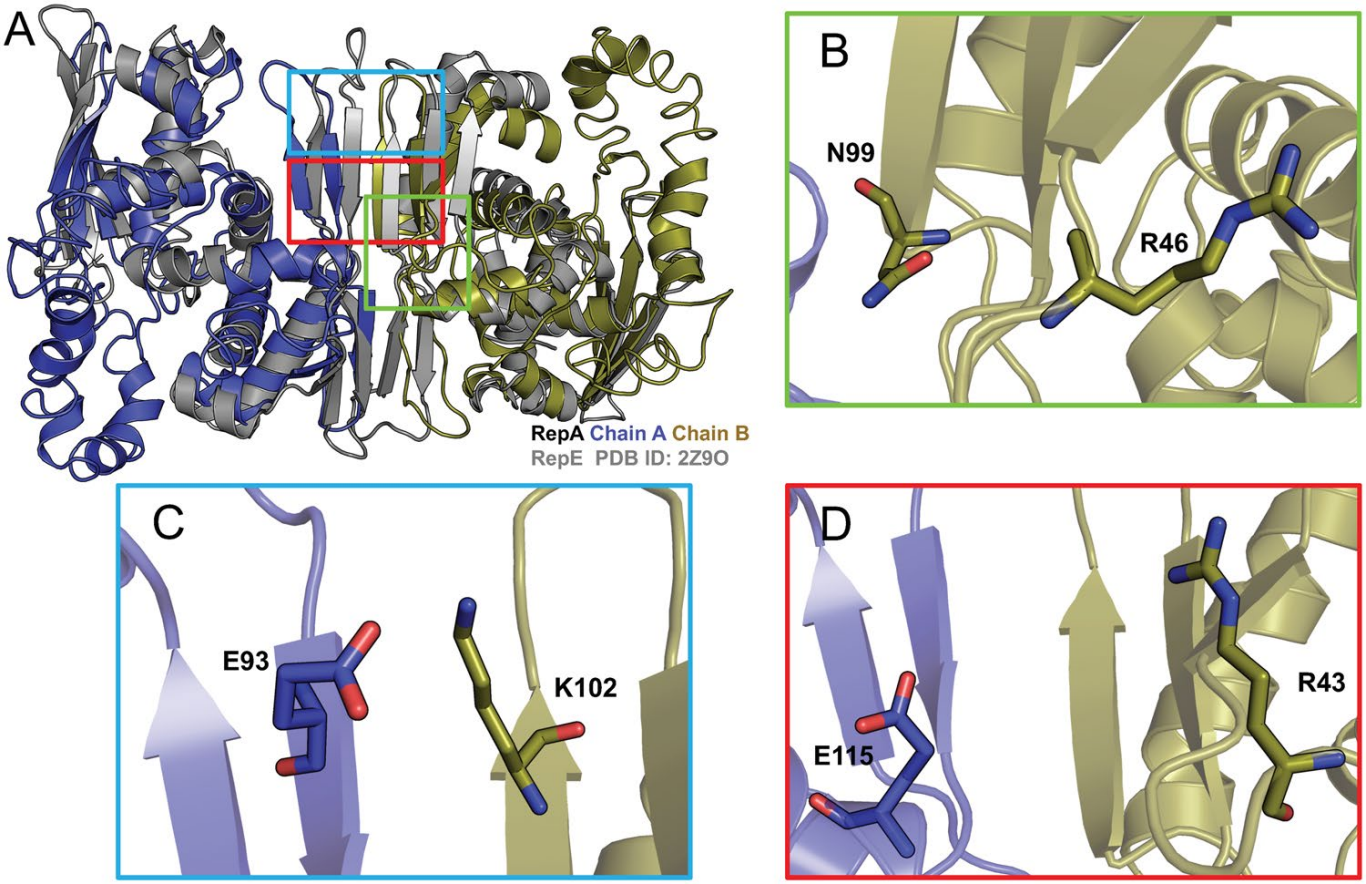
Homology model of RepA homodimer (shown in blue and gold) based on the RepE structure (shown in gray). (**A**) Full protein dimer. (**B**) Zoomed in view of N99 and R46 residues, the two most commonly isolated RepA mutants. (**C**) Potential electrostatic interaction between residues E93 and K102. (**D**) Potential electrostatic interaction between residues E115 and R43.

In order to infer the structural position of the mutated residues identified in our screen, we generated a homology model of the homodimer RepA based on the crystal structure of the homologous protein RepE, the protein that controls the replication of the mini-F plasmid (PDB ID: 2Z9O) (Fig. 3A)^7^. The RepE homodimer functions as a repressor while the monomer acts as a replication initiator. Though RepA shares only 19% sequence identity to RepE, the RepA homology model displayed a very similar fold with the RepE protein structure, having a root-mean-square deviation (RMSD) of 3.12 Å throughout the entire polypeptide backbone (Fig. 3A).

The majority of the identified mutations were located at or near the dimerization domain of RepA (Fig. 3B–D). Two of the most common mutations identified in our study, N99 and R46, were located close to the intermolecular β-sheets near the dimerization interface (Fig. 3B and Supplementary Figure 3). A potential interaction can be observed between N99 residue from monomer A and the neighboring N99 residue from monomer B (Supplementary Figure 3). In addition, several other mutants point to potential electrostatic interactions at the dimer interface. Residues E93 and K102 are located at the intermolecular β-sheets in the dimerization interface and are close enough for potential electrostatic integrations (Fig. 3C). A similar scenario can be observed between E115 and R43 (Fig. 3D). All the charged residues mentioned are consistent with residues that are also present in the RepE dimerization interface^7^. In RepE, the dimerization interface is mainly formed by intermolecular β-sheets with several key interactions of charged residues. A similar residue interaction is observed in the RepA homology model. Based on these observations, the majority of the mutants that we isolated appear likely to be involved in dimerization, either through stabilizing secondary structure at the dimerization interface, or through direct electrostatic interactions.

### Mutations at the RepA dimerization interface result in a variety of plasmid copy numbers

**Figure 4:**
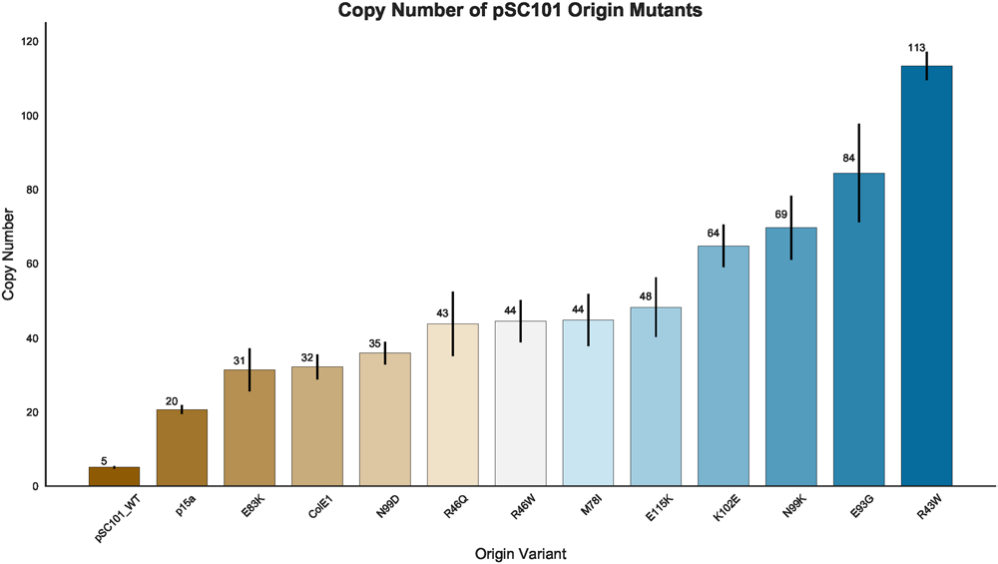
Copy number of control plasmids and pSC101 RepA mutants as determined by qPCR. Error bars represent 95% CI (n = 3).

Of the 14 unique mutated amino acid residues within RepA that we identified in our screen, only one has been rigorously characterized– E93^10^. Peterson *et al*. showed that an E93K mutation yielded a copy number of ~30 copies/cell, while an E93R mutation resulted in a copy number of ~240 copies/cell^10^. To probe how our newly identified mutations impacted copy number, we created 10 amino acid substitutions at 8 amino acid residues within the RepA coding sequence of plasmid pBbS8k-RFP, chosen either by the frequency of occurrence in our screen or predicted location at the dimerization interface based on our homology model. The pBbS8k-RFP plasmid is a Bglbrick family plasmid that contains a pSC101 origin, as well as the gene encoding the red fluorescence protein (RFP) under the control of an *araBAD* promoter^1^. The copy number of wild-type pBbS8k-RFP, in addition to that of all ten of our selected mutated variants, was determined by qPCR in the absence of arabinose. Additionally, the copy number of pBbA8k-RFP and pBbE8k-RFP, containing a p15a and ColE1 origin, respectively, were also calculated. All ten mutations tested showed increased copy numbers ranging from ~31 to ~113 copies/cell (Fig. 4). Mutations E83K and N99D yielded copy numbers of ~31 and ~35 copies/cell, respectively, similar to that of the ColE1 origin of replication (~32 copies/cell). Mutations R46Q, R46W, M78I, and E115K all had roughly equivalent copy numbers of approximately 45 copies/cell, while mutations K102E, N99K, E93G, and R43W all yielded higher copy numbers ranging from ~64 to ~113 copies/cell (Fig. 4).

We observed, as have other groups^10^, that the nature of the substitution within RepA can dramatically affect the resulting copy number. Two mutations at N99, N99D and N99K, resulted in markedly different copy numbers (~35 and ~69 copies/cell). Similarly, previous work has shown that mutations E93K and E93R raise the copy number to ~30 and ~240 copies/cell, respectively, while a E93G mutation resulted in a copy number of ~84 copies/cell^10^. However, in some cases multiple mutations at the same residue yielded similar copy numbers, such as mutations R46Q and R46W, which both resulted in pSC101 variants with ~45 copies/cell. From these results, it appears that a wide range of copy numbers can be obtained from a variety of *repA* mutations. However, whether or not these origin variants result in useful protein expression remained to be determined.

### Copy number in pSC101 *repA* mutants does not correlate with expression of RFP

**Figure 5:**
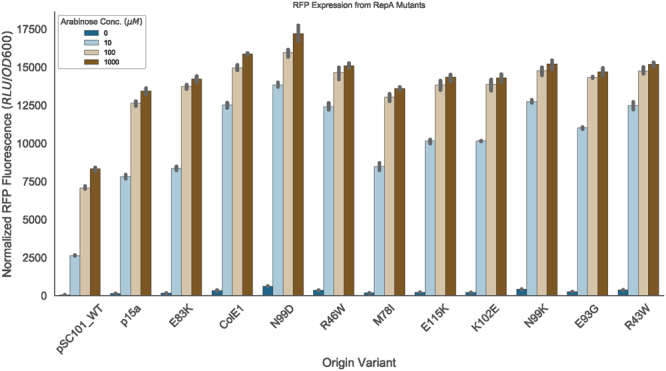
Production of RFP as a function of plasmid copy number and inducer concentration. Expression of RFP as measured by fluorescence normalized to OD_600_ following induction from the *araBAD* promoter with increasing concentrations of arabinose induction. Error bars represent 95% CI (n = 3). Plasmids are ordered by increasing copy number.

Though previous work has characterized the copy number of pSC101-derived mutants, the effect these mutations have on heterologous protein expression has not been similarly tested^10^. To better understand this connection, we performed a series of fluorescence protein expression assays to gauge whether copy number was correlated with increased RFP production from our vectors. To achieve this, we transformed our mutant and wild-type plasmids into *E*. *coli* DP10, which has the arabinose transporter, encoded by *araFGH*, under control of a constitutive promoter^15^ to allow for inducible expression from the *araBAD* promoter, and evaluated expression of RFP at increasing concentrations of arabinose (Fig. 5). As expected, all mutants had higher levels of RFP expression than wild-type pSC101 at all concentrations of arabinose tested. However, there was no overall correlation between copy number and RFP expression at any concentration of arabinose (Supplementary Figure 4). In fact, the highest expression of RFP achieved was from the N99D mutant, which has a copy number of ~35 copies/cell, similar to that of the ColE1 origin. Of the mutants with copy numbers below 35 copies/cell, mutant E83K (copy number of ~31 copies/cell) showed slightly higher expression than the wild-type p15a origin, which had a copy number of ~20 copies/cell. These two mutated origins, N99D and E83K, may prove useful in future metabolic engineering efforts.

### Mutant pSC101 origins remain compatible with other commonly used plasmid origins of replication

**Figure 6:**
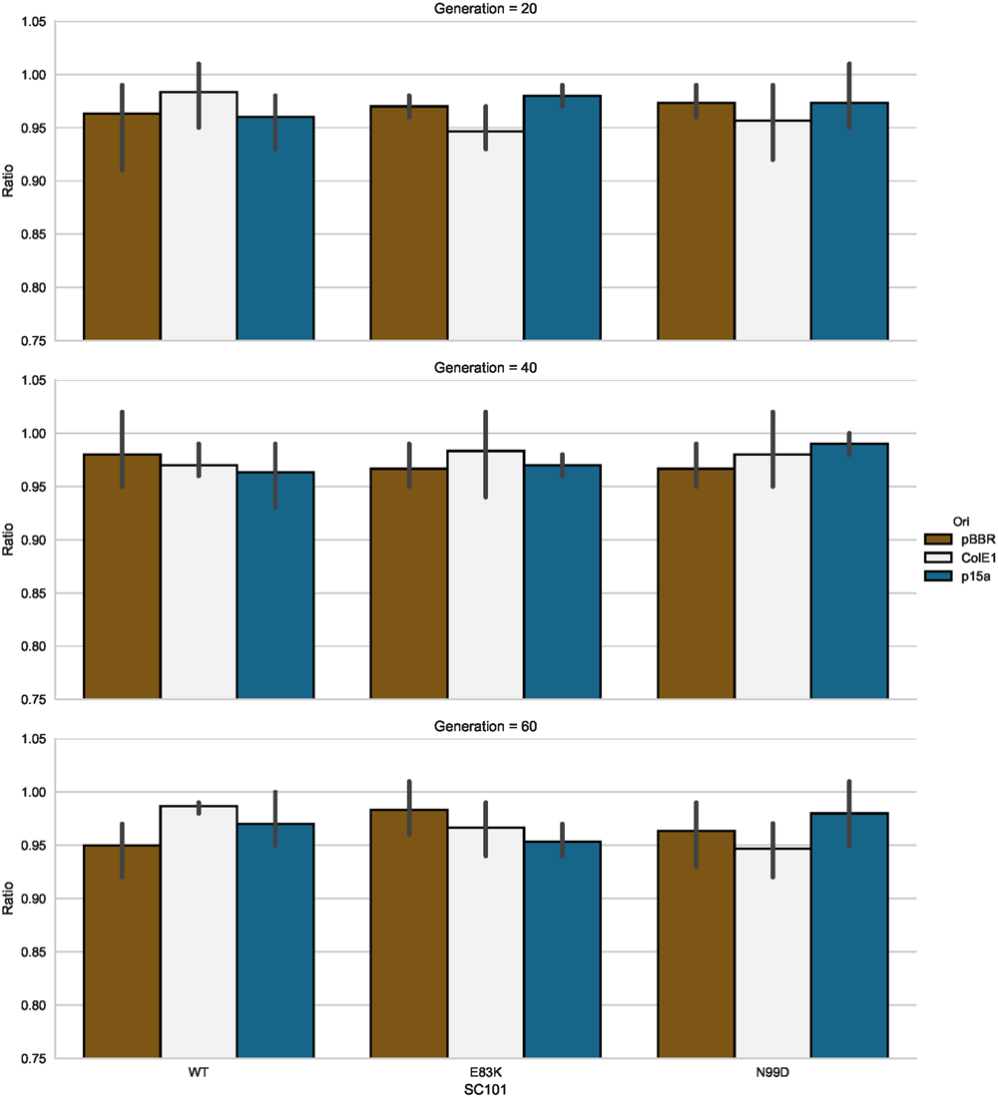
Plasmid compatibility of potentially useful pSC101 origin mutants with other commonly used *E*. *coli* plasmid origins. pSC101 wt and the E83K and N99D mutants encoded kanamycin resistance, while pBBR and p15a encoded carbenicillin resistance. Ratios of the number of CFUs recovered on Carb/Kan LB plates relative to the number of CFUs recovered on LB Carb alone. Error bars represent 95% CI.

In order for higher copy variants of the pSC101 origin to be useful in synthetic biology, they must maintain their compatibility with other commonly used origins of replication. Previous work has demonstrated that specific high-copy pSC101 mutants maintained compatibility with the pBBR origin, but did not investigate other common origins^10^. To interrogate compatibility more thoroughly used a previously described method wherein we transformed either a pSC101 wild-type vector or the N99D and E83K mutants, all of which encoded the kanamycin resistance marker, into strains of *E*. *coli* harboring either p15a, pBBR, or ColE1 origins of replication with a carbenicillin selectable marker^10^. Cells containing both plasmids were passaged for 60 generations while only selecting for the carbenicillin resistance marker. Compatibility was assessed every 20 generations by determining the ratio of cells that survived plating on LB Carb/Kan plates relative to LB Carb plates (Fig. 6). All pSC101 origins were maintained at greater than 93% of all cells for all origins tested, indicating that both the N99D and the E83K mutants retain compatibility with other commonly used *E*. *coli* origins.

### Mutations predicted to be at the RepA dimer interface can be combined to affect copy number

**Figure 7:**
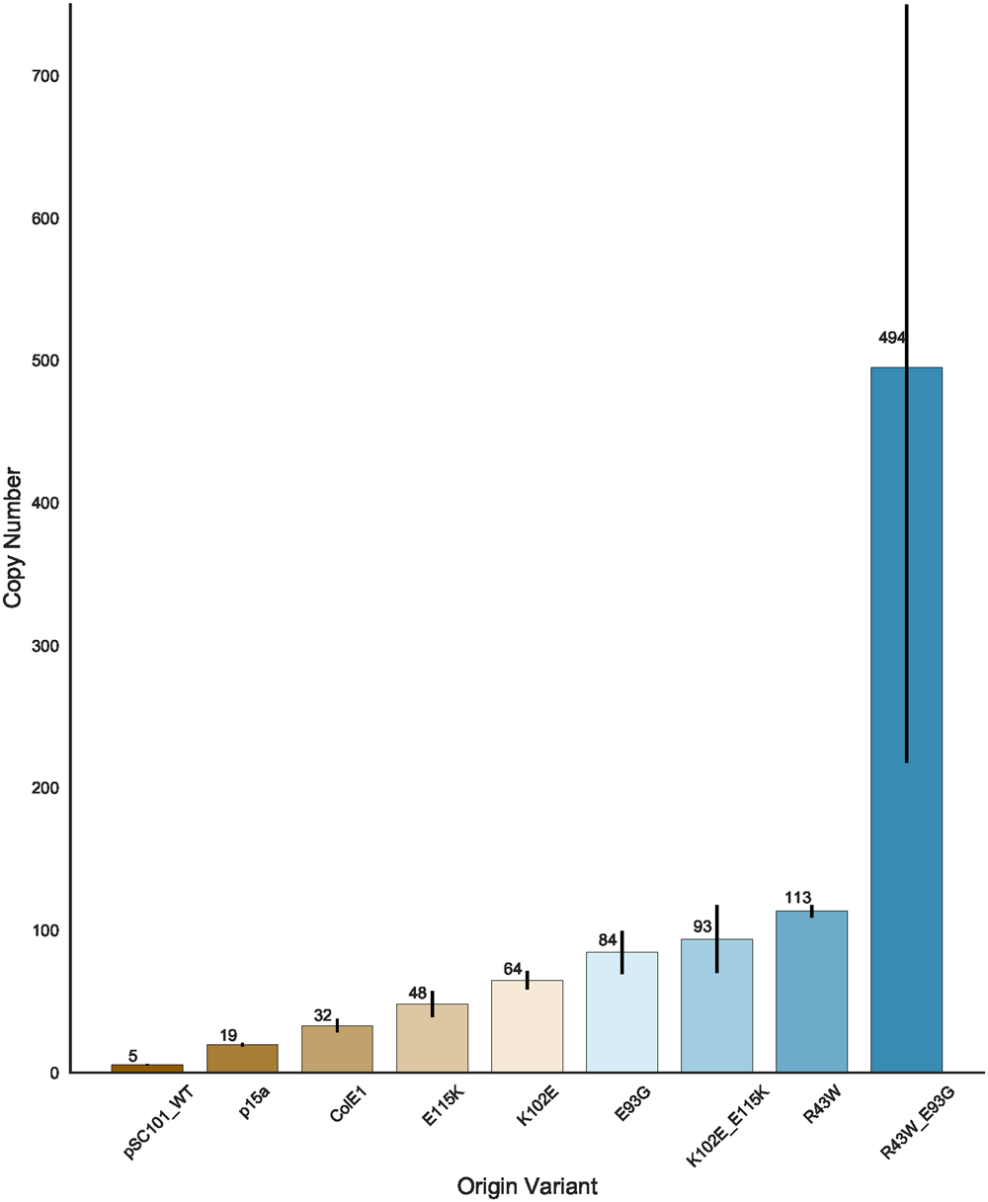
Copy number of control plasmids, parent RepA mutations, and double RepA mutations predicted to be involved in electrostatic interactions between monomers, as estimated by qPCR. Error bars represent 95% CI (n = 3).

While there may be limited benefit to increasing the copy number of the pSC101 origin past ~35 copies/cell, we were still interested in knowing if mutations at multiple loci could be combined additively to affect copy number. Based on our homology modelling, we identified two pairs of amino acids that could potentially be involved in electrostatic interactions at the dimerization interface, E93 and K102, as well as E115 and R43. We sought to disrupt both of these interactions by creating two double mutants, K102E-E115K and R43W-E93G. As before, we determined the copy number of the double mutants by qPCR (Fig. 7). The copy number of the K102E-E115K double mutant was roughly equivalent to the sum of the copy number of its individual component mutations. However, the R43W-E93G mutant had a much higher copy number of ~494 copies/cell, more than two and a half times that of its corresponding single mutants combined. This suggests that these two mutations in tandem are sufficient to severely disrupt the ability of RepA to dimerize.

While the copy number did dramatically increase in double mutant RepA proteins, neither of the double mutants was able to produce as much RFP at high arabinose concentration (100 μM and 1000 μM) as the N99D single mutation; nonetheless, the R43W-E93G mutant was able to produce RFP at levels greater than that of the ColE1 origin of replication at 10 μM arabinose (Supplementary Figure 5A). At higher concentrations of arabinose induction both double mutants had reduced growth rates, particularly in R43W-E93G (Supplementary Figure 6A). Though there was no correlation between fluorescence and copy number within our RepA mutants, there appeared to be a correlation between copy number and maximal growth rate as arabinose concentration increased with a Pearson-R of −0.91 at 100 μM arabinose and −0.87 at 1000 μM arabinose (Supplementary Figures 6B–E).

## Discussion

In this work, we isolated 10 previously undescribed RepA mutations that increase the copy number of the pSC101 origin. Single mutations resulted in copy numbers ranging from ~31 to ~113 copies/cell, compared to ~5 copies/cell in wild-type pSC101 plasmids. The majority of these mutations are in charged amino acids that are predicted to lie on the dimerization interface of the RepA protein. Two of these mutations, R43W and E93G, could be combined to achieve a copy number of ~494 copies/cell. When these mutations were incorporated into a plasmid containing RFP under the control of an arabinose-inducible promoter, no overall correlation was observed between the copy number and fluorescence. As copy number increased, there was a corresponding decrease in growth rate, with the highest copy double mutant showing severely diminished growth at higher levels of arabinose induction.

Homology modelling suggests that many of the mutations identified in this study lie within β-sheets on the dimerization interface of the RepA protein. Structural studies of the RepA homolog, RepE which controls the copy number of the mini-F plasmid, indicated a very similar intermolecular β-sheet dimeric interface^16^. RepE plays an important role in controlling replication of the F plasmid origin, ori2. Monomeric RepE binds to ori2 to initiate replication, whereas RepE homodimers act as an autogenous repressor by binding to the *repE* operator^16^. In another study, RepA was activated by the heat shock proteins DnaK and DnaJ^17^. Both DnaK and DnaJ activated RepA by converting RepA dimers into monomers in an ATP-dependent reaction and simultaneously activated oriP1 DNA binding^17^. Based on these observations, the majority of the mutants that we isolated appear to be involved in dimerization, either through stabilizing the secondary structure at the dimerization interface, or through direct electrostatic interactions. Each mutation in the double mutant constructs R43W-E93G or K102E-E115K is located at different regions of the dimer interface and may explain the additive effect of increase copy number. Such structural perturbations in RepA may cause a disruption in its oligomeric state and hence, plasmid copy regulation. Due to the limits of homology models and lack of a RepA crystal structure, it is difficult to reliably categorize and measure distinct residue interactions at the dimer interface. Future efforts in obtaining a crystal structure of RepA may provide further detail.

Of the mutations that we isolated in this work, E83K and N99D may be particularly useful for synthetic biology applications. Plasmids harboring the E83K mutation showed a very similar expression profile to that of a p15a expression vector, while the N99D mutant plasmid demonstrated slightly higher expression than the ColE1 derivative. Importantly, both of these mutants retain compatibility with other commonly used origins of replication in *E*. *coli*. Previous work has demonstrated that copy number mutants maintain their compatibility with ColE1 origin plasmids, making these mutants potentially valuable when engineering systems that require multiple medium or high copy vectors^10^. RFP expression, as measured by fluorescence, plateaued for RepA mutant-derived plasmids with copy numbers above ~35 copies/cell, which can be explained in a variety of ways. While very high-copy double mutants achieved higher expression at low levels of induction (10 µM), they were severely detrimental to growth at higher inducer concentration. One limitation of this study was the reliance on RFP fluorescence as a measure of expression, which may not be predictive of other proteins^18,19^. Future work will need to be conducted to assess the expression profiles of other proteins. The reasons for this plateau may be manifold due to mRNA stability, metabolic burden of maintaining the plasmid, or limitation of key intracellular metabolites^4,20^. Further work will be necessary to fully characterize why no additional expression can be obtained by increasing copy number in these systems.

As metabolic engineering and synthetic biology mature as disciplines, many groups have looked beyond model organisms as chasses for engineering efforts^21^. While rudimentary genetic systems exist for some of these organisms, they lack the robust tool sets that have been developed for model organisms such as *Saccharomyces cerevisiae* and *E*. *coli*. With relatively few high- or medium-copy vectors characterized for non-model organisms, we believe that this work could serve as a template for developing differential copy vectors derived from plasmids controlled by RepA homologs in non-model hosts. By mutagenizing *repA* or a *repA* homolog within a plasmid expressing a resistance determinant from an inducible promoter, variable copy numbers should be able to be isolated by imposing different strengths of selection for the resistance determinant. Alternatively, further structural information on RepA and its homologs may allow for more targeted engineering of dimer interactions that would allow for predictable copy numbers as a function of the strength of dimerization. Regardless of the approach taken, mutagenesis of RepA-dependent plasmids may offer a relatively easy way to develop a range of useful copy number variants in hosts that are desirable for metabolic engineering.

## Methods

### Media, Bacterial Growth Conditions, and Reagents

All bacterial strains are listed in Table 1. All strains and plasmids created in this work are available through the public instance of the JBEI registry (https://public-registry.jbei.org/folders/338). Bacteria were cultivated in Luria-Bertani (LB) Miller media (BD Biosciences, San Jose, CA) at 37 °C unless otherwise noted, supplemented with kanamycin (50 mg/L, Sigma Aldrich, USA) or spectinomycin (100 mg/L, Fisher Scientific, USA) when indicated. Tetracycline hydrochloride used in plate selections was added at concentrations indicated, as were inducers bromocyclohexane, caprolactam, δ-undecalactone, and γ-nonalactone. All compounds were purchased through Sigma Aldrich, USA.

**Table 1:** All strains and plasmids used in this study.

Strain	Notes	Reference	JBEI ID
*E*. *coli* DH10B		Invitrogen	
*E*. *coli* DP10	∆*araFGH* ∆P-*araE* pCP18-araE: for linear arabinose induction	^15^	
*E*. *coli* XL1-Red	Commercial mutator strain	Agilent	
**Plasmid**	**Notes**	**Reference**	
pBbS8k-RFP_R43W	RepA Mutant, Kan	This work	JPUB_009625
pBbS8k-RFP_R46W	RepA Mutant, Kan	This work	JPUB_009627
pBbS8k-RFP_R46Q	RepA Mutant, Kan	This work	JPUB_009629
pBbS8k-RFP_M78I	RepA Mutant, Kan	This work	JPUB_009631
pBbS8k-RFP_E83K	RepA Mutant, Kan	This work	JPUB_009633
pBbS8k-RFP_E93G	RepA Mutant, Kan	This work	JPUB_009635
pBbS8k-RFP_N99D	RepA Mutant, Kan	This work	JPUB_009637
pBbS8k-RFP_N99K	RepA Mutant, Kan	This work	JPUB_009639
pBbS8k-RFP_K102E	RepA Mutant, Kan	This work	JPUB_009641
pBbS8k-RFP_E115K	RepA Mutant, Kan	This work	JPUB_009643
pBbS8k-RFP_R43W_E93G	RepA Double Mutant, Kan	This work	JPUB_009645
pBbS8k-RFP_K102E_E115K	RepA Double Mutant, Kan	This work	JPUB_009647
pBbS8k-RFP	AraC Expression Vector, pSC101 Ori, Kan	^1^	
pBbA8k-RFP	AraC Expression Vector, p15a Ori, Kan	^1^	
pBbE8k-RFP	AraC Expression Vector, ColE1 Ori, Kan	^1^	
pBbE8a-RFP	AraC Expression Vector, ColE1 Ori, Amp	^1^	
pBbB8a-RFP	AraC Expression Vector, BBR Ori, Amp	^1^	
pBbS8a-RFP	AraC Expression Vector, p15a Ori, Amp	^1^	
pMGT1	ChnR Biosensor with TetA Reporter, pSC101 Ori, Spec	This work	JPUB_009650
pBbSlactamC-mCherry (star)	ChnR Biosensor with mCherry Reporter, pSC101 Ori, Spec	^11^	

Strains and plasmids created in this study can be found at https://public-registry.jbei.org/folders/338.

### DNA Manipulation

All plasmids used in this study are listed in Table 1. All primers used in this study are listed in Supplementary Table 1. Plasmids were designed using Device Editor and Vector Editor software^22,23^, and primers for construction of plasmids were designed using j5^24^. All plasmids were assembled via Gibson Assembly^25^. Plasmids were isolated using the Qiaprep Spin Miniprep kit (Qiagen, USA), and all primers were purchased from Integrated DNA Technologies (IDT, Coralville, IA).

### Site Directed Mutagenesis of *repA*

To construct site-specific mutants, the parent plasmid pBbS8k-RFP was used as a template to amplify the entire plasmid into two PCR products. Mutations were introduced in primers that bound within the *repA* gene. Plasmids were assembled from these products via Gibson assembly, and mutations were confirmed via Sanger sequencing (Quintara Biosciences, Albany, CA).

### Creation of a Mutagenesis Library of pMGT1

Mutagenesis of the lactam-biosensing pMGT1 *tetA* reporter plasmid was performed *in vivo* in the *E*. *coli* mutator strain XL1-Red (Agilent Technologies, Santa Clara, CA). The plasmid was transformed into XL1-Red competent cells via electroporation and plated on LB agar plates supplemented with spectinomycin. After incubating at 37 °C overnight, ~2000 colonies were scraped and were then resuspended in 50 mL of LB medium supplemented with spectinomycin and grown overnight at 37 °C. Afterward, 1 mL of the culture was removed and stored at −80 °C for future plasmid isolation. This overnight was then used to inoculate 500 µL of LB spectinomycin 1:100 in each well of a 96-well deep-well plate (VWR, USA). Passaging of cultures was done in a 96-well format to help prevent selective sweeps by non-mutator phenotypes of XL1-Red that may arise. Passages were performed for five days every 20 hours, by inoculating fresh media in 96-well plates via a pin-replicator. Each day after passaging, culture from all wells was pooled and frozen for subsequent plasmid isolation. Plasmids from all passages were isolated and pooled and then transformed into *E*. *coli* MegaX DH10B T1R Electrocomp Cells (Invitrogen, Carlsbad, CA) via electroporation, yielding roughly twelve million transformants. These transformants were pooled and frozen at −80 °C for later use.

### Selection for Mutants

To select for mutations that were more sensitive to ligands of interest, *E*. *coli* DH10B cultures containing either the mutagenized plasmids or the parent plasmid were grown overnight in LB medium supplemented with spectinomycin, and then plated on LB agar plates that contained various concentrations of inducers and tetracycline. Inducers used to select for mutations were bromocyclohexane, caprolactam, δ-undecalactone, and γ-nonalactone. Plates contained either 1000 µM, 500 µM, 100 µM, or 0 µM of each inducer. Plates contained either 50 mg/L, 25 mg/L, 12.5 mg/L, or 0 mg/L tetracycline (see Supplementary Figure 2). Colonies from library plates that were able to grow on higher concentrations of tetracycline at lower concentrations of inducer after overnight incubation were considered for further analysis. Thirty plasmids selected from each inducer were purified and re-sequenced to identify the location of mutations.

### Re-sequencing Plasmids via Next-Generation Sequencing

Nextera libraries were constructed using the Illumina Nextera XT Library Preparation Kit and the Nextera XT Index Kit v2 (Illumina, San Diego, CA). Liquid transfers were carried out on the BioMek NX, Biomek Fx (Beckman Coulter, Indianapolis, IN), and Labcyte Echo 550 acoustic liquid dispensing system (Labcyte, Sunnyvale, CA). During tagmentation, DNA was fragmented by transposases, and transposon terminal sequences were appended as adapters. Unique barcode combinations were added during PCR amplification. The tagmentation and PCR amplification steps were performed with several modifications to the standard Nextera protocol: 1) all steps were performed in a single 384-well PCR plate, 2) the tagmentation reaction volume was reduced to 1 µL using the Echo 550, and 3) a heat-kill step (15 minutes at 70 °C) was added to circumvent the need for purification after tagmentation. For the qPCR step, 7.5 µl SsoAdvanced Universal SYBR Green Supermix (2×) (Bio-Rad, Hercules, CA) and 5.5 µl Nuclease free water (Thermo Fisher Scientific, Waltham, MA) were added to the tagmentation reaction. The end point fluorescence values from qPCR on a CFX384 (Bio-Rad, Hercules, CA) were used to normalize concentrations using the Echo 550. This enabled Ampure XP bead purification (A63880, Beckman Coulter, Indianapolis, IN) of the pooled library in a single tube. The purified library was quantified using the Qubit DNA HS assay (Invitrogen, Carlsbad, CA), and the fragment size profile was determined using the Bioanalyzer 2100 (Agilent Technologies, Santa Clara, CA). Sequencing was performed on the MiSeq (Illumina, San Diego, CA) using 2 × 300 cycles. MiSeq reads were demultiplexed using the embedded MiSeq Reporter (MSR) software. Read mapping and variant calling were performed using BWA^26^, and GATK^27^.

### Isolation of Total DNA

Isolation of total DNA was performed via the method employed by Lee *et al*., with minor changes^1,28,29^. Overnight cultures harboring plasmids of interest were inoculated 1:100 in LB kanamycin. Cultures were grown to an OD_600_ of ~0.3, and then 2 mL of culture was pelleted and frozen at –80 °C. Cell pellets were resuspended in 400 μL of 50 mM Tris/50 mM EDTA, pH 8, and then permeabilized with 8 μL of 50 mg/mL lysozyme (Sigma Aldrich, USA) in 10 mM Tris/1 mM EDTA, pH 8, followed by incubation at 37 °C for 30 minutes. Cells were then lysed by the addition of 8 μL of 5% SDS and 8 μL of 20 mg/mL Proteinase K solution (Qiagen, USA), after which they were mixed with a syringe with a 21-gauge, 1.5-inch needle, followed by incubation at 50 °C for 30 minutes. After inactivating Proteinase K by incubation at 75 °C for 10 min, 2 μL of 100 mg/mL RNase A solution (Qiagen, USA) was added and then incubated at 37 °C for 30 minutes. Total DNA was extracted via a phenol:chloroform extraction, followed by ethanol precipitation as described previously^1^. Isolated gDNA was digested for 6 hours at 37 °C with Fast Digest EcoRI (Thermo Fisher Scientific, Waltham, MA).

### Estimation of Copy Number via qPCR

Estimation of copy number was also carried out in a manner similar to that of Lee *et al*., with minor changes^1,8,28^. Primer sets specific to the neomycin phosphotransferase II (*nptII*) gene (forward: GCGTTGGCTACCCGTGATAT, reverse: AGGAAGCGGTCAGCCCAT) and 16 S rDNA gene (forward: CCGGATTGGAGTCTGCAACT, reverse: GTGGCATTCTGATCCACGATTAC) were used for real-time qPCR. *E*. *coli* DH10B gDNA was used for calibrating the 16 S rDNA primers, while *E*. *coli* BW25113 with a single copy of *nptII* integrated into the genome was used to calibrate the *nptII* primers. Real-time qPCR was conducted on a BioRad CFX with 96-well reaction blocks in the presence of SYBR Green under the following conditions: 1 × ssoAdvance SYBR Green Supermix (BioRad, Hercules, CA) and 150 nM *nptII* or 500 nM 16 S primers, in 20 μL reactions. Real-time qPCR cycling parameters were 95 °C for 3 minutes, followed by 40 cycles of 30 seconds at 95 °C, and 45 seconds at 60 °C. Threshold cycles (Ct) were determined with CFX Manager (BioRad, Hercules, CA) software for all samples.

### Growth Rate and Fluorescence Assays

Growth rates of bacterial strains were estimated through a microplate reader kinetic assay. Strains were grown overnight from glycerol stocks in LB liquid medium with kanamycin, then diluted 1:100 into fresh LB media with kanamycin amended with either 1000 µM, 100 µM, 10 µM, or 0 µM arabinose in 96-well plates (Falcon, 353072). Plates were sealed with a gas-permeable microplate adhesive film (VWR, USA), and then optical density and fluorescence were monitored for 22 hours in an Infinite F200 Pro (Tecan Life Sciences, San Jose, CA) plate reader at 30 °C. Optical density was measured at 600 nm, while fluorescence was measured using an excitation wavelength of 575 nm and an emission wavelength of 620 nm with a manually set gain of 35. In between reads, the plate was shaken at a linear amplitude of 6 mm.

To measure RFP production from plasmids, fluorescence measurements were obtained from single time points of cells grown in deep-well 96-well plates. Cells were grown in 500 µL of LB medium with kanamycin supplemented with either 1000 µM, 100 µM, 10 µM, or 0 µM arabinose. Plates were sealed with AeraSeal film (Excel Scientific, AC1201-02) and grown for 22 hours at 30 °C on a 200 rpm shaker rack. After incubation, 100 µL from each well was aliquoted into a black, clear-bottom 96-well plate and fluorescence was measured with an Infinite F200 Pro (Tecan Life Sciences, San Jose, CA) plate reader. Optical density was measured at 600 nm (OD_600_), while fluorescence was measured using an excitation wavelength of 575 nm and an emission wavelength of 620 nm with a manually set gain of 35.

### Plasmid Compatibility Assay

Mutant plasmids pBbS8k-RFP_E83K and pBbS8k-RFP_N99D were cotransformed into *E*. *coli* DH10B with a BglBrick family plasmid containing the gene encoding RFP under the control of an *araBAD* promoter, and a carbenicillin resistance cassette. The origin of replication for the latter vector was either p15a, ColE1, or pBBR1. To test plasmid stability and compatibility, the transformants were grown overnight in LB containing 100 µg/mL carbenicillin. Each overnight culture was considered to be the result of 10 generations of growth. Each overnight culture was diluted by a factor of 1:1,000 (3 μL into 3 mL) and grown again for a total of 60 generations, i.e. six days. Every other day, after 20 generations of growth, the cultures were diluted 1:50,000 and plated onto LB plates containing only carbenicillin, as well as plates containing both carbenicillin and kanamycin. Colonies were then counted after incubation overnight. Stability was assessed as the ratio between the number of colonies present on the carb/kan plates and the number of colonies present on the carb only plates.

### RepA Structural Modelling

The RepA homology model was generated using the structure prediction software I-TASSER (https://zhanglab.ccmb.med.umich.edu/)^30,31^. The internal C-score scoring function in I-TASSER was utilized to select the best homology model. The known homodimeric RepE protein structure (PDB ID: 2Z9O) was utilized to generate a homodimeric structure of the RepA homology model. The RepA homodimeric model was energy minimized using CHIMERA^32^. All figures related to the RepA homology model were visualized and generated by PyMOL (The PyMOL Molecular Graphics System, Version 1.8 Schrödinger, LLC.).

### Data Visualization and Statistical Analysis

All data were analyzed using custom Python scripts. All graphs were visualized using either Seaborn or Matplotlib. Calculation of 95% confidence intervals was conducted via the Scipy library.

## Electronic supplementary material

Supplementary Information

## References

[1] Lee TS (2011). BglBrick vectors and datasheets: A synthetic biology platform for gene expression. J Biol Eng.

[2] George KW (2014). Correlation analysis of targeted proteins and metabolites to assess and engineer microbial isopentenol production. Biotechnol Bioeng.

[3] Alonso-Gutierrez J (2013). Metabolic engineering of Escherichia coli for limonene and perillyl alcohol production. Metab Eng.

[4] Carrier T, Jones KL, Keasling JD (1998). mRNA stability and plasmid copy number effects on gene expression from an inducible promoter system. Biotechnol Bioeng.

[5] Furuno S, Watanabe-Murakami Y, Takebe-Suzuki N, Yamaguchi K (2000). Negative control of plasmid pSC101 replication by increased concentrations of both initiator protein and iterons. J Gen Appl Microbiol.

[6] Chattoraj DK (2000). Control of plasmid DNA replication by iterons: no longer paradoxical. Mol Microbiol.

[7] Nakamura A, Wada C, Miki K (2007). Structural basis for regulation of bifunctional roles in replication initiator protein. Proc Natl Acad Sci USA.

[8] Wadood A, Dohmoto M, Sugiura S, Yamaguchi K (1997). Characterization of copy number mutants of plasmid pSC101. J Gen Appl Microbiol.

[9] Giraldo R, Fernández-Tresguerres ME (2004). Twenty years of the pPS10 replicon: insights on the molecular mechanism for the activation of DNA replication in iteron-containing bacterial plasmids. Plasmid.

[10] Peterson J, Phillips GJ (2008). New pSC101-derivative cloning vectors with elevated copy numbers. Plasmid.

[11] Zhang J (2017). Development of a Transcription Factor-Based Lactam Biosensor. ACS Synth Biol.

[12] Steigedal M, Valla S (2008). The Acinetobacter sp. chnB promoter together with its cognate positive regulator ChnR is an attractive new candidate for metabolic engineering applications in bacteria. Metab Eng.

[13] Chae TU, Ko Y-S, Hwang K-S, Lee SY (2017). Metabolic engineering of Escherichia coli for the production of four-, five- and six-carbon lactams. Metab Eng.

[14] Dietrich JA, Shis DL, Alikhani A, Keasling JD (2013). Transcription factor-based screens and synthetic selections for microbial small-molecule biosynthesis. ACS Synth Biol.

[15] Pitera DJ, Paddon CJ, Newman JD, Keasling JD (2007). Balancing a heterologous mevalonate pathway for improved isoprenoid production in Escherichia coli. Metab Eng.

[16] Ishiai M, Wada C, Kawasaki Y, Yura T (1994). Replication initiator protein RepE of mini-F plasmid: functional differentiation between monomers (initiator) and dimers (autogenous repressor). Proc Natl Acad Sci USA.

[17] Wickner S, Hoskins J, McKenney K (1991). Monomerization of RepA dimers by heat shock proteins activates binding to DNA replication origin. Proc Natl Acad Sci USA.

[18] Reisbig MD, Hossain A, Hanson ND (2003). Factors influencing gene expression and resistance for Gram-negative organisms expressing plasmid-encoded ampC genes of Enterobacter origin. J Antimicrob Chemother.

[19] Strack RL (2008). A noncytotoxic DsRed variant for whole-cell labeling. Nat Methods.

[20] Smolke CD, Keasling JD (2002). Effect of copy number and mRNA processing and stabilization on transcript and protein levels from an engineered dual-gene operon. Journal of Biochemical and Microbiological Technology and Engineering.

[21] Loeschcke A, Thies S (2015). Pseudomonas putida-a versatile host for the production of natural products. Appl Microbiol Biotechnol.

[22] Chen J, Densmore D, Ham TS, Keasling JD, Hillson NJ (2012). DeviceEditor visual biological CAD canvas. J Biol Eng.

[23] Ham TS (2012). Design, implementation and practice of JBEI-ICE: an open source biological part registry platform and tools. Nucleic Acids Res.

[24] Hillson, N. J., Rosengarten, R. D. & Keasling, J. D. j5 DNA assembly design automation software. *ACS Synth Biol* **1**, 14–21 (2012). 10.1021/sb2000116 23651006

[25] Gibson DG (2009). Enzymatic assembly of DNA molecules up to several hundred kilobases. Nat Methods.

[26] Li, H. Aligning sequence reads, clone sequences and assembly contigs with BWA-MEM. *arXiv* (2013).

[27] McKenna A (2010). The Genome Analysis Toolkit: a MapReduce framework for analyzing next-generation DNA sequencing data. Genome Res.

[28] Mason G, Provero P, Vaira AM, Accotto GP (2002). Estimating the number of integrations in transformed plants by quantitative real-time PCR. BMC Biotechnol.

[29] Pushnova EA, Geier M, Zhu YS (2000). An easy and accurate agarose gel assay for quantitation of bacterial plasmid copy numbers. Anal Biochem.

[30] Roy A, Kucukural A, Zhang Y (2010). I-TASSER: a unified platform for automated protein structure and function prediction. Nat Protoc.

[31] Zhang Y (2008). I-TASSER server for protein 3D structure prediction. BMC Bioinformatics.

[32] Pettersen EF (2004). UCSF Chimera–a visualization system for exploratory research and analysis. J Comput Chem.

