# Supplementary material for: Isolation and characterization of novel mutations in the pSC101 origin that increase copy number
Source: Sci Rep. 2018 Jan 25;8:1590. doi: 10.1038/s41598-018-20016-w (PMC5785507; doi:10.1038/s41598-018-20016-w)
Supplement: Supplementary file 1 — Supplementary Information [file 41598_2018_20016_MOESM1_ESM.pdf]

Isolation and characterization of novel mutations in the pSC101 origin that increase copy number

Mitchell G. Thompson[1,2,3,#], Nima Sedaghatian[1,2,#], Jesus F. Barajas[2,4], Maren Wehrs[1,2], Constance B. Bailey[1,2], Nurgul Kaplan[1,2], Nathan J. Hillson[1,2,4], Aindrila Mukhopadhyay[1,2], and Jay D. Keasling[1,2,5,6,7*]

[1]DOE Joint BioEnergy Institute, 5885 Hollis Street, Emeryville, CA 94608, USA.

[2]Biological Systems & Engineering Division, Lawrence Berkeley National Laboratory, Berkeley, CA 94720, USA.

[3]Department of Plant and Microbial Biology, University of California, Berkeley, CA 94720, USA

[4]DOE Agile BioFoundry, Emeryville, CA 94608, USA

[5]Department of Bioengineering, University of California, Berkeley, CA 94720, USA

[6]Department of Chemical and Biomolecular Engineering, University of California, Berkeley, CA 94720, USA

[7]The Novo Nordisk Foundation Center for Biosustainability, Technical University of Denmark, Denmark, Building 220 Kemitorvet, 2800Kgs Lyngby Denmark

[#]These authors contributed equally to this work.

Supplementary Information

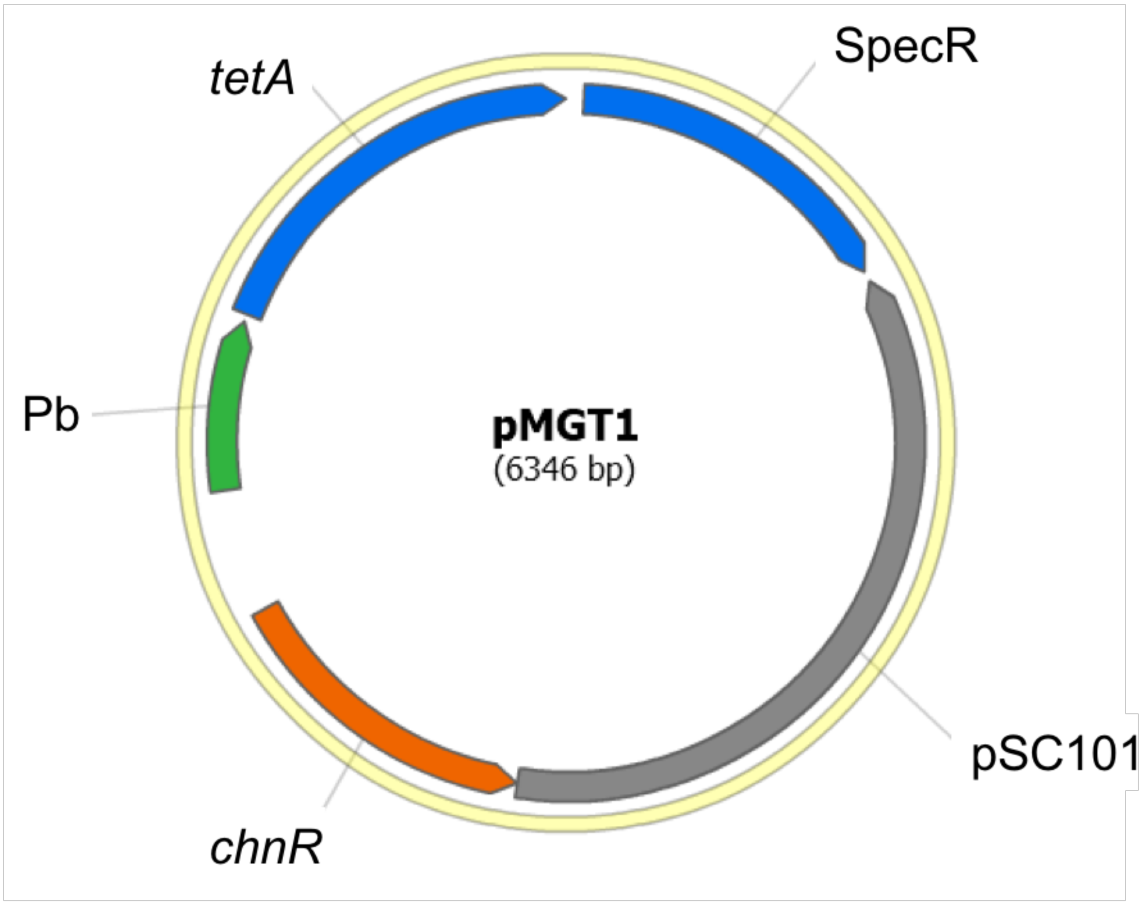

Supplementary Figure 1: Plasmid map of pMGT1. The tetracycline resistance gene *tetA* is under the control of the *chnR* responsive promoter Pb, with a pSC101 origin of replication.

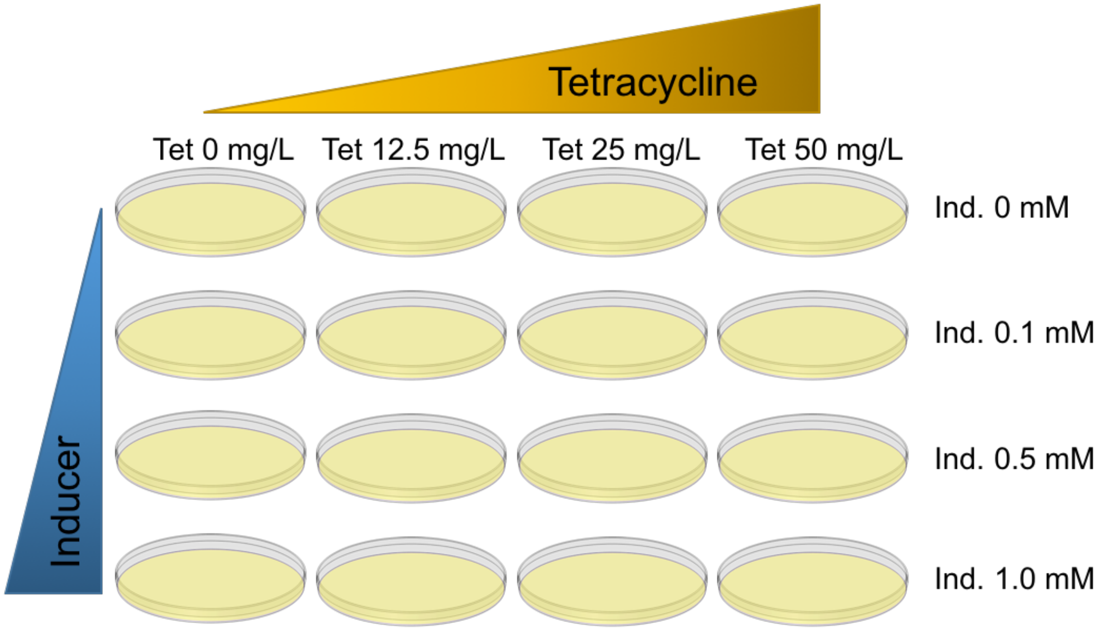

Supplementary Figure 2: Experimental design of plate-based checkerboard assay for the selection of mutants more sensitive to inducer. Mutants more sensitive to a given ligand should grow on higher concentrations of tetracycline with lower concentrations of inducer. Inducers tested were caprolactam, bromocyclohexane, γ-nonalactone, and δ-undecalactone.

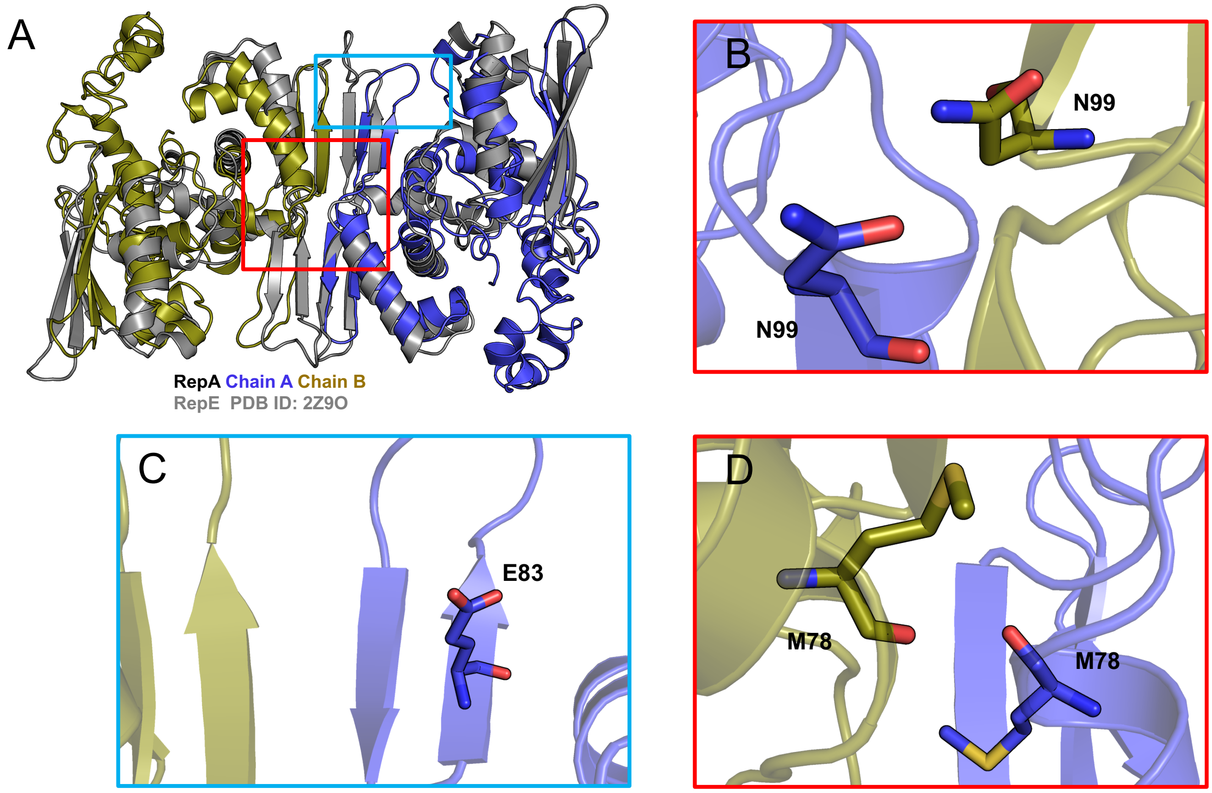

Supplementary Figure 3: A) Homology model of RepA protein based on RepE crystal structure with other commonly isolated mutations highlighted. B) Potential homointeraction of residue N99. C) Location of residue E83 within the secondary structure of the putative dimerization interface. D) Location of residue M78 within potential secondary structure of the dimerization interface.

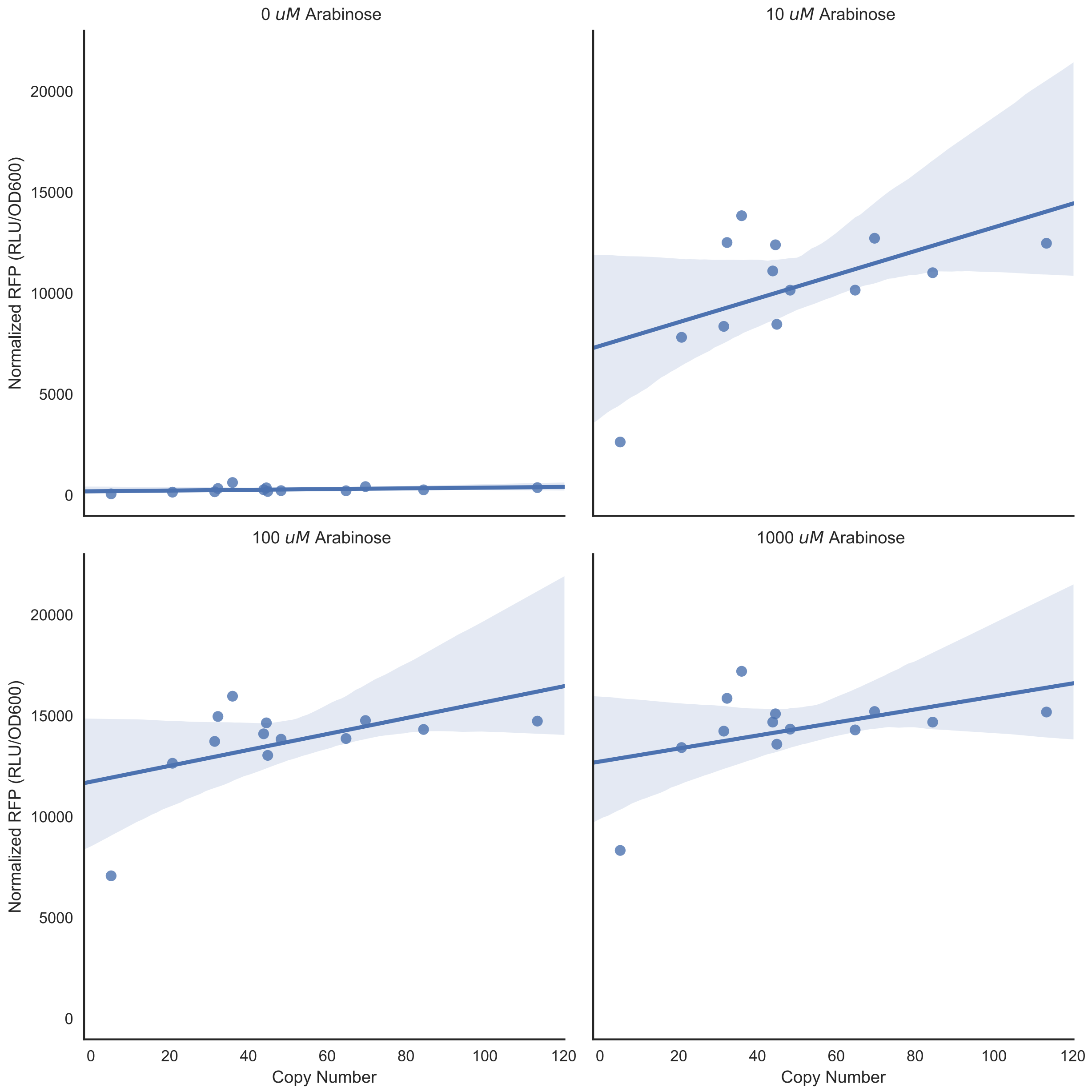

Supplementary Figure 4: Correlation of copy number to RFP fluorescence normalized to OD600 in control plasmids and single *repA* mutants. Shaded area represents 95% CI.

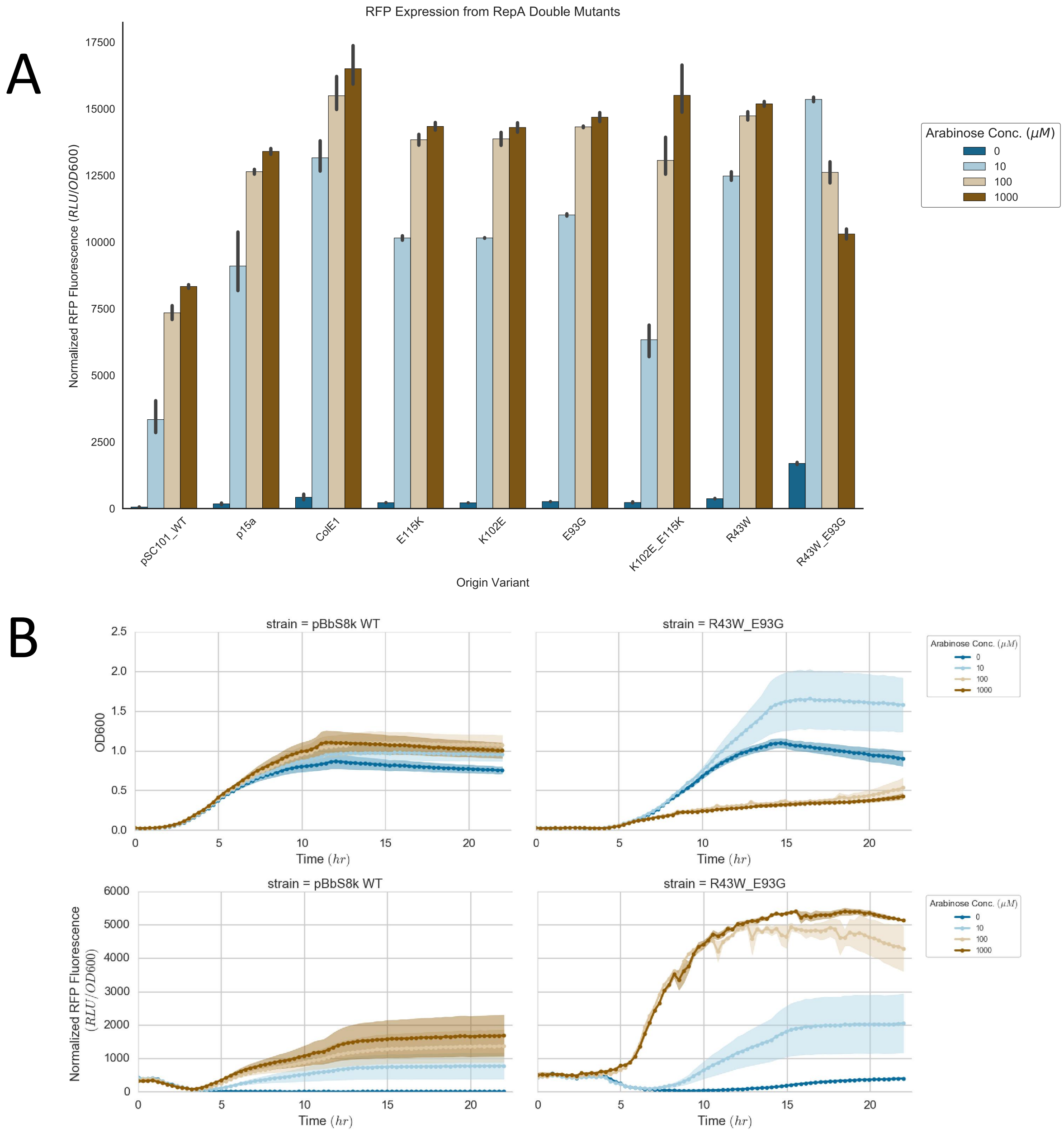

Supplementary Figure 5: A) Maximal RFP fluorescence normalized to OD600 of control plasmids, parent *repA* mutations, and double *repA* mutations predicted to be involved in electrostatic interactions between monomers. Error bars represent 95% CI (n=3). Plasmids are ordered by increasing copy number. B) Growth curves showing OD600 (Top) and RFP fluorescence normalized to OD600 (Bottom) of lowest and highest copy number plasmids identified (pSC101_WT and R43W_E93G, respectively).

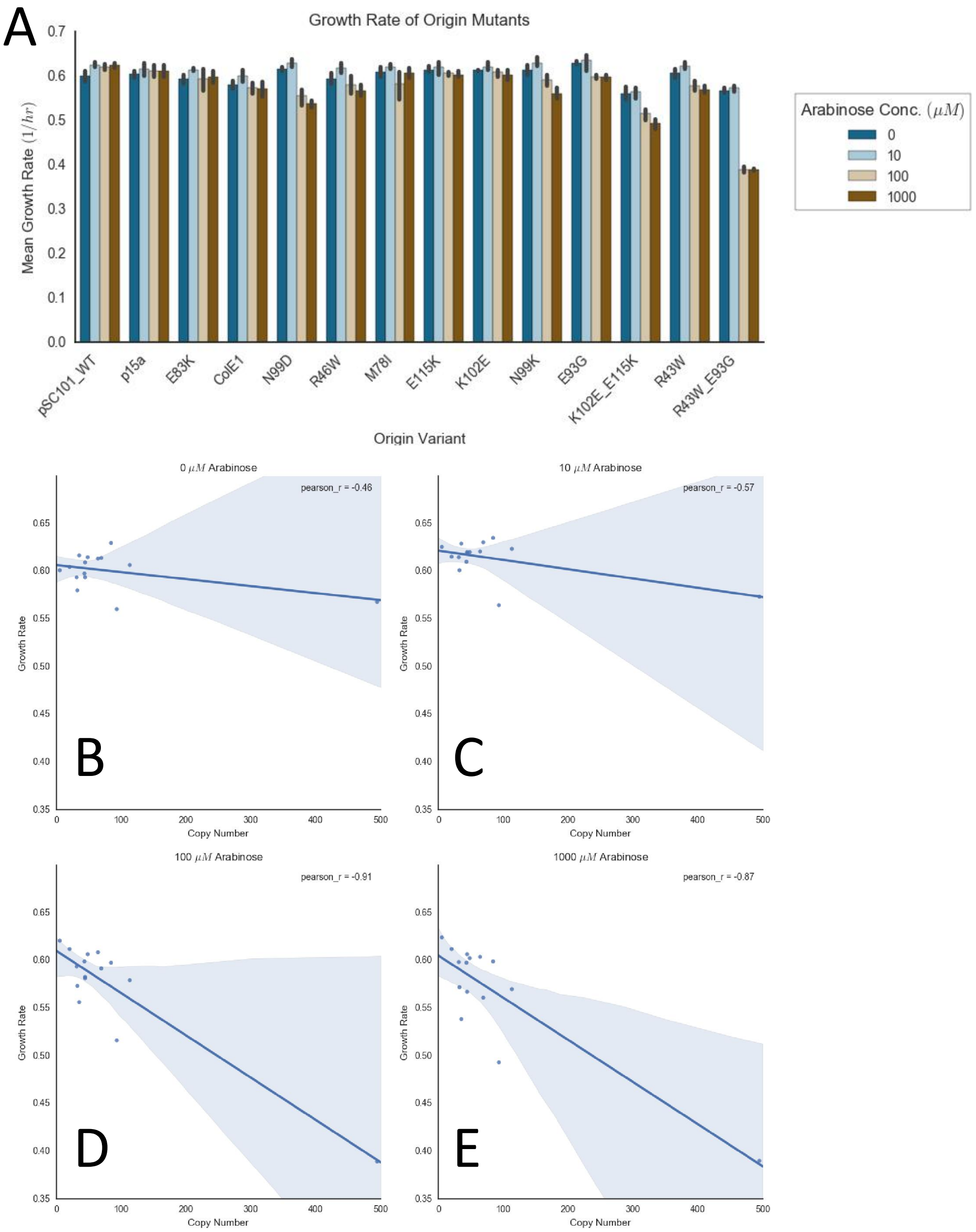

Supplementary 6: (A) Growth rate of *E. coli* DH10B carrying control plasmids and pBbS8k-RFP with RepA mutants. Error bars represent 95% CI (n=3). (B-E) Correlation of plasmid copy number and maximal growth rate of control plasmids at 0uM, 10uM, 100uM, and 1000uM arabinose. Shaded area represents 95% CI.

**Name**	**Sequence**	**Notes**
317_RepA_C136T_R	aacgataccgtccattctttc	pBbS8k-RFP_R46W construction, used with kan_F
316_RepA_C136T_F	gaaagaatggacggtatcgtt	pBbS8k-RFP_R46W construction, used with kan_R
318_RepA_A295G_R	ttccagtggacagactatgcc	pBbS8k-RFP_N99D construction, used with kan_F
319_RepA_A295G_F	ggcatagtctgtccactggaa	pBbS8k-RFP_N99D construction, used with kan_R
325_RepA_G137A_F	ggaaagaacagacggtatcgttc	pBbS8k-RFP_R46Q construction, used with kan_F
326_RepA_G137A_R	acgataccgtctgttctttcc	pBbS8k-RFP_R46Q construction, used with kan_R
327_RepA_C297G_F	cagtggacaaagtatgccaag	pBbS8k-RFP_N99K construction, used with kan_F
328_RepA_C297G_R	cttggcatactttgtccactg	pBbS8k-RFP_N99K construction, used with kan_R
332_RepA_C278G_F	aggctttgggattttccagtgg	pBbS8k-RFP_E93G construction, used with kan_F
329_RepA_C278G_R	ccactggaaaatcccaaagcct	pBbS8k-RFP_E93G construction, used with kan_R
330_RepA_A304G_F	caaactatgccgagttctcaagc	pBbS8k-RFP_K102E construction, used with kan_F
331_RepA_A304G_R	gcttgagaactcggcatagtttg	pBbS8k-RFP_K102E construction, used with kan_R
a127t_F	cacgattgaaaaccctacatggaaagaacggacgg	pBbS8k-RFP_R43W_E93G construction from R43W, used with kan_R
a127t_R	ccgtccgttctttccatgtagggttttcaatcgtg	pBbS8k-RFP_R43W_E93G construction from R43W, used with kan_F
g234a_F	ttccacagttctcgttatcagctctctggttgctt	pBbS8k-RFP_M78I construction, used with kan_R
g234a_R	aagcaaccagagagctgataacgagaactgtggaa	pBbS8k-RFP_M78I construction, used with kan_F
g247a_F	ccaaaggattcctgattttcacagttctcgtcatcag	pBbS8k-RFP_E83K construction, used with kan_R
g247a_R	ctgatgacgagaactgtgaaaatcaggaatcctttgg	pBbS8k-RFP_E83K construction, used with kan_F
g343a_F	gaattagtttttagtgaaaagatattgccttatcttttcc	pBbS8k-RFP_K102E_E115K construction from E115K, used with kan_R
g343a_R	ggaaaagataaggcaatatcttttcactaaaaactaattc	pBbS8k-RFP_K102E_E115K construction from E115K, used with kan_F
kan_F	ctgatgctcttcgtccagatcatcctgatc	Used to construct all site-directed mutants
kan_R	gaagtgccggggcaggatctcctgtcatctc	Used to construct all site-directed mutants
271_OriSeqFpSC101	tagtaattatcattgactagcc	site-directed mutant sequencing primer
j5_00063_(tetA)_forward	cgtaatgagggtaccatgaaacccaacataccccctgatcgt	pDVA01657_TetA_PlusSpacer construction
j5_00064_(tetA)_reverse	gcacgatcaacggttagcgatcggctcgttgccc	pDVA01657_TetA_PlusSpacer construction
j5_00061_(biosensor_backbone)_forward	cgctaaccgttgatcgtgctatgatcgac	pDVA01657_TetA_PlusSpacer construction
j5_00062_(biosensor_backbone)_reverse	gtttcatggtaccctccattacgacatgtgaatttattc	pDVA01657_TetA_PlusSpacer construction
nphII forward	GCGTTGGCTACCCGTGATAT	qPCR of nphII
nphII reverse	AGGAAGCGGTCAGCCCAT	qPCR of nphII
nphII forward	CCGGATTGGAGTCTGCAACT	qPCR of 16S rDNA
nphII reverse	GTGGCATTCTGATCCACGATTAC	qPCR of 16S rDNA

Supplementary Table 1: All primers used in this study
